# HOXB2 promotes cisplatin resistance by upregulating lncRNA DANCR in ovarian cancer

**DOI:** 10.1186/s13048-024-01424-1

**Published:** 2024-06-08

**Authors:** Xiao Li, Zhen Zheng, Wanzhen Zhou, Huixian Huang, Yang Zhou, Qinyang Xu, Xiaolu Zhu, Yincheng Teng

**Affiliations:** 1 Department of Obstetrics and Gynecology, Shanghai Jiao Tong University Affiliated Sixth People’s Hospital, Shanghai Jiao Tong University School of Medicine, Shanghai, 200233 China; 2 Fujian Maternity and Child Health Hospital College of Clinical Medicine for Obstetrics & Gynecology and Pediatrics, Fujian Medical University, Fujian, China

**Keywords:** Ovarian cancer, Cisplatin resistance, HOXB2, DANCR, ABC transporter

## Abstract

Ovarian cancer (OV) is a highly fatal malignant disease that commonly manifests at an advanced stage. Drug resistance, particularly platinum resistance, is a leading cause of treatment failure because first-line systemic chemotherapy primarily relies on platinum-based regimens. By analyzing the gene expression levels in the Cancer Genome Atlas database, Genotype-Tissue Expression database, and Gene Expression Omnibus datasets, we discerned that *HOXB2* was highly expressed in OV and was associated with poor prognosis and cisplatin resistance. Immunohistochemistry and loss-of-function experiments on *HOXB2* were conducted to explore its role in OV. We observed that suppressing *HOXB2* could impair the growth and cisplatin resistance of OV in vivo and in vitro. Mechanical investigation and experimental validation based on RNA-Seq revealed that *HOXB2* regulated ATP-binding cassette transporter members and the ERK signaling pathway. We further demonstrated that *HOXB2* modulated the expression of long non-coding RNA DANCR, a differentiation antagonizing non-protein coding RNA, and thus influenced its downstream effectors *ABCA1*, *ABCG1*, and ERK signaling to boost drug resistance and cancer proliferation. These results verified that high expression of *HOXB2* correlated with platinum resistance and poor prognosis of OV. Therefore, targeting *HOXB2* may be a promising strategy for OV therapy.

**Supplementary Information:**

The online version contains supplementary material available at 10.1186/s13048-024-01424-1.

## Introduction

Ovarian cancer (OV) is a highly malignant gynecological cancer with an unsatisfactory survival rate because of the diagnostic and therapeutic challenges. For over twenty years, surgery followed by platinum-based systemic chemotherapy has been the first-line treatment approach [1, 2]. However, platinum resistance leads to treatment failure and incurable relapse [3], rendering it the most disturbing impediment in OV research and clinical practice.

Homeobox genes (HOX genes) are transcription factors containing a sequence of 183 nucleotides that encode a 61-amino acid DNA-binding domain called the homeodomain [4]. They were initially identified to support spatial body development in fruit flies and were proved to be highly conserved in animals [5, 6]. Members of this family are fundamental information carriers that guide the vertebrate formation and shape animal morphology. Aberrant HOX gene expression has been demonstrated to promote oncogenesis through various mechanisms, including suppression of differentiation, driving tumor growth, and anti-apoptosis [7]. As a member of the HOX gene superfamily, the transcription factor *HOXB2* has also been reported to function in malignancies, including lung cancer, and Wilms tumor [8–10]. A study on OV has established that *HOXB2* can be an effective cancer marker for prognosis and can indicate the severity of inflammatory infiltration in high-risk tumors [11]. Another integrative analysis of genome-wide association studies identified a HOX-centric network containing *HOXB2* associated with OV risk [12]. Therefore, the *HOXB2* function in OV must be elucidated.

Long non-coding RNAs (lncRNA) regulate gene expression primarily by acting as molecular sponges for miRNAs. Differentiation antagonizing non-protein coding RNA (DANCR) on chromosome 4q12 [13] was first identified to suppress progenitor differentiation within the epidermis [14] and was demonstrated to increase cancer cell stemness in hepatocarcinoma [15]. DANCR is a multifunctional lncRNA related to carcinogenesis that has been documented to sequester dozens of tumor-suppressing miRNAs and activate signaling pathways, including PI3K/AKT, MAPK/ERK, and JAK/STAT [16, 17]. Research on OV indicates that DANCR promotes angiogenesis by modulating the miR-145/VEGF axis [17]. Studies on drug resistance have reported that DANCR induces cisplatin tolerance in glioma and triple-negative breast cancer [18, 19], which drew our interest in this lncRNA.

This study aimed to identify upregulated *HOXB2* levels in OV and their correlation with platinum resistance and poor prognosis. We further investigated the molecular mechanisms of *HOXB2* in OV chemoresistance and proliferation through its regulatory function on DANCR. Therefore, *HOXB2* may serve as a therapeutic target for OV treatment.

## Materials and methods

### Clinical samples

Tissue microarray (TMA) comprising 117 samples from OV cases was purchased from Wuhan Servicebio Technology Co., Ltd., and 47 OV samples were collected from patients who underwent cisplatin and paclitaxel chemotherapy at Shanghai Sixth People’s Hospital. All patients provided informed consent before sampling. This study was approved by the Research Ethics Committee of Shanghai Sixth People’s Hospital, affiliated with Shanghai Jiao Tong University School of Medicine.

### Cell culture

Cisplatin-resistant A2780/DDP cells, human OV cell lines A2780, and OVCAR8 were preserved in the State Key Laboratory of Oncogenes and related genes, Shanghai Cancer Institute. The cells were cultured in Dulbecco’s modified Eagle’s medium with 10% fetal bovine serum and 100 µg/mL penicillin/streptomycin (P/S) at 37 °C in a 5% CO_2_ atmosphere. A 10 mM Cisplatin (HY-17,394, MCE) was dissolved in sterile ddH_2_O and added to the culture medium when required.

### Cell transfection

Short hairpin RNAs targeting *HOXB2* and a randomized sequence were constructed into a GV112 vector (Genechem). Cells were infected with 1 × 10^6^ recombinant lentivirus-transducing units and 5 µg/mL polybrene. After 72 h, 5 µg/mL puromycin was applied to exclude uninfected cells. The sequences were as follows:

shNC: TTCTCCGAACGTGTCACGT.

sh*HOXB2*-1: GCTCATGATCTGGACGTGAAA.

sh*HOXB2*-2: CCACGTCAAGATTTCTGATTT.

Small interfering RNAs (siRNAs) were also transduced into cancer cells by jetPRIME transfection reagent (Polyplus, France) to knock down *HOXB2*. The sequences were as follows:

siNC: sense 5’-UUCUCCGAACGUGUCACGUTT-3’.

antisense 5’-ACGUGACACGUUCGGAGATT-3’.

si*HOXB2*-1: sense 5’-GGCAGGUCAAAGUCUGGUUTT-3’.

antisense 5’-AACCAGACUUUGACCUGCCTT- 3’.

si*HOXB2*-2: sense 5’-GCCUUUAGCCGUUCGCUUATT-3’.

antisense 5’-UAAGCGAACGGCUAAAGGCTT-3’.

For DANCR overexpression, full-length human DANCR was cloned into a GV712 vector (Genechem), which was then transfected into cells using a jetPRIME reagent.

### Half maximal inhibitory concentration (IC_50_) examination

A2780/DDP and OV8 cells were seeded and grown for 24 h in 96-well plates (5 × 10^3^ cells/well). The original culture medium was replaced with different concentrations of the cisplatin solution. After 48 h of incubation, cell viability was determined using CCK-8, and the drug IC_50_ was calculated using GraphPad Prism 8.0.

### Cell proliferation assay and plate colony formation assay

Different groups of cells were transferred to 96-well plates at a density of 1000 cells/well. Relative cell viability was detected at OD_450nm_ after incubation with cell counting kit-8 (CCK-8, B34304, Bimake) for 1 h for the next five days.

For the colony formation assay, thousand cells from each cell group were seeded in 6-well plates and grown with cisplatin (10 µM for A2780/DDP and 5 µM for OV8) or equal PBS for two weeks. The clones were then fixed with 4% paraformaldehyde and stained with crystal violet.

### Immunofluorescence and EdU assay

Cells from different groups were seeded in 15 µ slide 8-well plates (80,826, Ibidi). After treatment, cells were fixed with 4% paraformaldehyde, perforated with 0.1% Triton-X for 10 min, and blocked with 5% BSA for 30 min. Next, we incubated cell samples with anti-γH2A.X (1:100, GB111841, Servicebio) at 4 °C overnight and in the corresponding FITC-conjugated secondary antibody at room temperature for 1 h. Nuclei were then stained with DAPI for 10 min. A Confocal microscopy system was used for the image acquisition.

The EdU incorporation assay (KTA2030, Abbkine) was conducted following manual instructions to detect cell viability. Briefly, cells were co-cultured with 50 µM EdU for 2 h before being fixed and permeabilized. After the click reaction, cells were counterstained with DAPI and imaged by Confocal microscopy system. Likewise, cells were co-cultured with 50 µM EdU for 2 h and then be suspended, fixed, and peameabilized. After following click reaction, we used flow cytometry to quantify EdU-positive cells.

### Cell apoptosis assay

Cell apoptosis rates were quantified by flow cytometry using the Annexin V-FITC/PI apoptosis kit (E-CK-A211, Elabscience) according to the protocol after 24–48 h of cisplatin treatment (20 µM for A2780/DDP, and 10 µM for OV8).

### Protein extraction and western blotting

Cell lysates were extracted using RIPA lysis buffer (G2033, Servicebio). Total cell protein was normalized using the BCA method (23,227, Thermo Fisher) and separated using 8–12% SDS-PAGE gel electrophoresis before being transferred onto a nitrocellulose membrane. After blocking with 5% milk for 1 h, the membrane was incubated with primary antibodies overnight at 4 °C and secondary antibodies for 1 h at room temperature. Antibodies used were as follows: anti-HOXB2 (1:1000, AY2582, Abways), anti-Beta Actin (1:5000, AB0035, Abways), anti-Beta tubulin (1:5000, 10086-1-AP, Proteintech), anti-γH2A.X (1:1000, GB111841, Servicebio), anti-Chk2 (1:10000, CY5633, Abways), anti-phospho Chk2 Thr68 (CY8878, Abways), anti-Chk1 (1:1000, 2360, Cell Signaling Technology), anti-phospho Chk1 Ser345 ((1:1000, 2348, Cell Signaling Technology), anti-ABCA1 (1:1000, ab66217, Abcam), anti-ABCG1 (1:1000, 13578-1-AP, Proteintech), anti-phospho Erk1/2 Thr202/Tyr204 (1:2000, 4370, Cell Signaling Technology), and anti-Erk1/2 (1:1000, 4695, Cell Signaling Technology). Enhanced chemiluminescence was conducted using an HRP substrate (WBKLS0500, Merck) and visualized by a Bio-Rad imaging system.

### RNA extraction and quantitative real-time PCR (qPCR)

The total RNA of cells was extracted and reverse transcribed using RNA TRIzol reagent (9108, Takara) and PrimeScript RT reagent kit (RR037A, Takara) following the instructions. qPCR was conducted with SYBR Master Mix (B21703, Bimake) on a 7500 Real-time PCR system (Applied Biosystems) at the commonly recommended thermal cycling settings. The 2^(−ΔΔCt)^ method was used to calculate the relative mRNA expression level using the expression of 18 s as the reference gene. Primer sequences were as follows:

18s-F ATCACCATTATGCAGAATCCACG,

18s-R GACCTGGCTGTATTTTCCATCC;

*HOXB2*-F CGCCAGGATTCACCTTTCCTT.

*HOXB2*-R CCCTGTAGGCTAGGGGAGAG;

*ABCA1*-F CATCTGGTTCTATGCCCGCT,

*ABCA1*-R TCTGCATTCCACCTGACAGC;

*ABCA7*-F TCCTGACCTCTCTGTCCCG,

*ABCA7*-R GGAGCTGGACCGGCTGT;

*ABCB4*-F GAAAGGCCAGACACTAGCCC,

*ABCB4*-R ACCATCGAGAAGCACTGTCC;

*ABCC3*-F GCCAAGAGGAACTTGACCCC,

*ABCC3*-R GACCTGGATGTCTAGGCTGTG;

*ABCC5*-F GCAGGGGCGCAGGAAT,

*ABCC5*-R GCTGGTTCTCTCCCTCACAC;

*ABCD1*-F CATGTTCTACCACAGGCCCA,

*ABCD1*-R GTGATGGAGAGCAGGGCAAT.

*ABCG1*-F CGTGGGCCCAGTGACAG;

*ABCG1*-R CCCTTCGAACCCATACCTGAC,

DANCR-F CAGTGCCACAGGAGCTAGAG;

DANCR-R GCAGCCTGTCCCTAACAGAA;

### Mouse xenograft models

Subcutaneous xenograft models were constructed by subcutaneously implanting 2 × 10^6^ shNC or sh*HOXB2* A2780/DDP cells into female BALB/c nude mice (6–8 weeks old). Once visible xenografts were grown, the mice with different cells were randomly divided into PBS and CDDP (cisplatin treated) groups. Equal volumes of PBS and 5 mg/kg CDDP solution were injected intraperitoneally every three days. Three weeks later, the mice were euthanized with CO_2_, and the xenografts were measured and weighed before being fixed with 4% paraformaldehyde. All mouse experiments were approved by the Shanghai Sixth People’s Hospital Research Ethics Committee affiliated with the Shanghai Jiao Tong University School of Medicine.

### Immunohistochemistry and TUNEL assay

Immunohistochemistry (IHC) was routinely performed on paraffin sections following the instructions and observed under an optical microscope. The sections were dewaxed, rehydrated, and heated in a citrate-based solution for antigen retrieval. After blocking in 10% BSA, the sections were stained with the following primary antibodies: anti-HOXB2 (1:100, AY2582, Abways) and anti-Ki67 (GB111141, Servicebio), followed by incubation in HRP conjugated secondary antibodies. The sections were then reacted with DAB substrate and counterstained with hematoxylin before being dehydrated, mounted, and covered.

Terminal deoxynucleotidyl transferase dUTP nick-end labeling (TUNEL) assay was performed to detect apoptotic cells in paraffin sections of mouse xenografts using a TUNEL kit (GB1507-50T, Servicebio) following the manual instructions.

### Chromogenic in situ hybridization (CISH)

CISH was routinely performed on paraffin sections following the protocols. The sections were dewaxed, dehydrated, repaired, and digested using protease K. After pre-hybridization, the sections were incubated in hybridization solution containing probes. The sections were then blocked by rabbit serum, color developed, and counterstained the nucleus before sealing and imaging. In this article, we used five probes targeting DANCR. Probe sequences are as follows:

TGCGACGGGCGCACAAACCAGAGA;

GCACTTCCGCAGACGTAAGAGACG;

CTGCACGGACACGTGGTTGCTACA;

GGGTCAGCTGCATTGAGTTAGCGG;

TTCTCCACCAGTCGGAGGTGGCAG.

### Statistical analyses

Data are expressed as mean ± standard deviation. The survival time was analyzed using the Kaplan-Meier method, and the P-value was calculated using the log-rank test. The results of the correlation analyses between *HOXB2* and the target genes were described using Pearson’s correlation coefficient. The statistical significances between groups were calculated using the chi-square test, Student’s *t*-test, or ANOVA, as appropriate.

## Results

### High *HOXB2* expression correlates with platinum resistance and poor prognosis in patients with OV

**Fig. 1:**
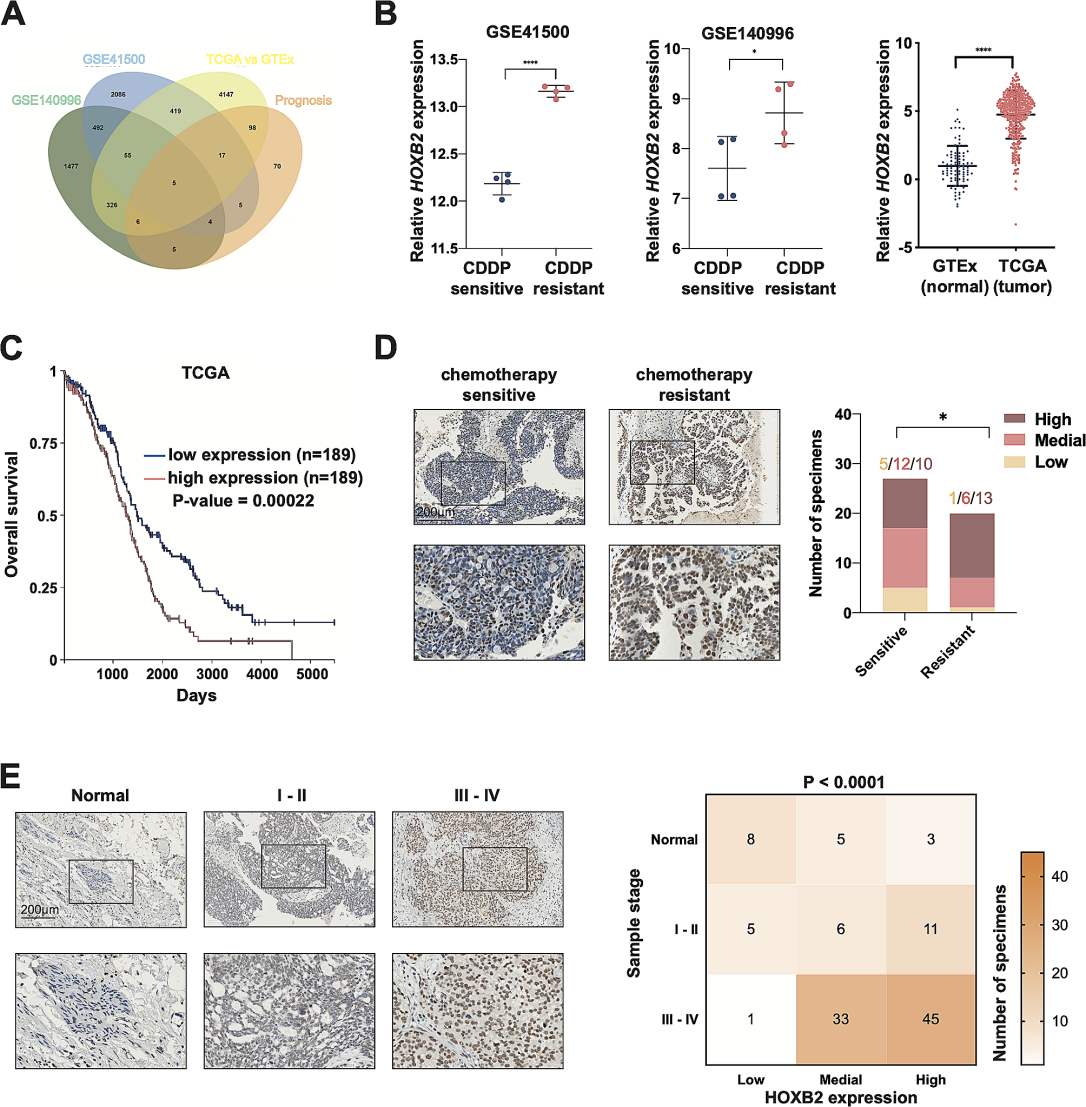
High *HOXB2* expression correlates with platinum resistance and poor prognosis in patients with OV. **A**. The target gene *HOXB2* was chosen from the intersection of the four gene clusters, as follows: **a**. Genes with higher expression in the TCGA OV dataset compared with the GTEx non-tumor dataset. **b**. Genes with predictive functions of prognosis based on TCGA OV dataset. **c**. Genes with higher expression in platinum-resistant OV cell lines than in platinum-sensitive cell lines in GSE140996. **d**. Genes with higher expression in platinum-resistant samples than in platinum-sensitive samples in GSE41500. **B**. Detail mRNA level of *HOXB2* in GSE41500, GSE140996, TCGA (*n* = 428), and GTEx (*n* = 88) datasets. A two-tailed P-value was calculated using an unpaired *t*-test. **C**. The overall survival curve for patients with OV was based on the expression of *HOXB2* in the TCGA dataset. A two-sided log-rank test was performed to calculate the statistical significance. **D**. Representative images (left) and statistical analysis (right) of IHC staining for HOXB2 in chemotherapy-resistant samples and chemotherapy-sensitive samples. Scale bar, 200 μm. The chi-square test was used for the analysis. **E**. Representative images (left) and statistical analysis (right) of IHC staining for HOXB2 in 117 OV TMA samples with different stages. Scale bar, 200 μm. The P-value was calculated with the chi-square test. *P < 0.05, **P < 0.01, ***P < 0.001, ***P < 0.0001

To identify potential target genes that influence the development and drug resistance of OV, we first analyzed the gene expression of patients with OV from the Cancer Genome Atlas (TCGA) public database and healthy individuals’ specimens from the Genotype Tissue Expression (GTEx) database. We obtained a cluster of highly expressed genes in OV compared to normal tissues (Fig. 1A: TCGA) and a cluster of genes related to the prognosis of OV (Fig. 1A: prognosis). Additional analysis of differentiated expressed genes (DEGs) from two datasets related to cisplatin resistance was done in the Gene Expression Omnibus (GEO) database. The GSE140996 dataset showed the gene expression levels of platinum-sensitive cancer cell lines and platinum-resistant cell lines. A cisplatin-sensitive cell line PEO1 from OV patients and a cisplatin-resistant cell line PEO4 from the same patient after 10 months of cisplatin chemotherapy were isolated for sequencing analysis in the GSE41500 dataset [20]. Considering the intersection of the aforementioned four gene clusters, we selected the gene HOXB2 as the target gene for this research (Fig. 1A).

The *HOXB2* expression was significantly higher in cancer tissues than in normal tissues. Compared to cisplatin-sensitive cases, the mRNA levels of *HOXB2* in drug-resistant OV samples were increased (Fig. 1B). Survival analysis based on the clinical information of patients with OV in TCGA indicated that *HOXB2* positively correlates with poor prognosis (Fig. 1C). Further histochemical staining of the OV tissue microarray was performed to confirm the high HOXB2 expression in cancer samples at the protein expression level (Fig. 1E). Additionally, OV specimens from Shanghai Sixth People’s Hospital were collected and tested by IHC. The proportion of highly expressed HOXB2 in cisplatin-resistant patients was higher than that in the cisplatin-sensitive groups (Fig. 1D). These results revealed that *HOXB2* expression is upregulated in OV and is associated with poor prognosis and cisplatin resistance.

### *HOXB2* promotes the OV cell growth

**Fig. 2:**
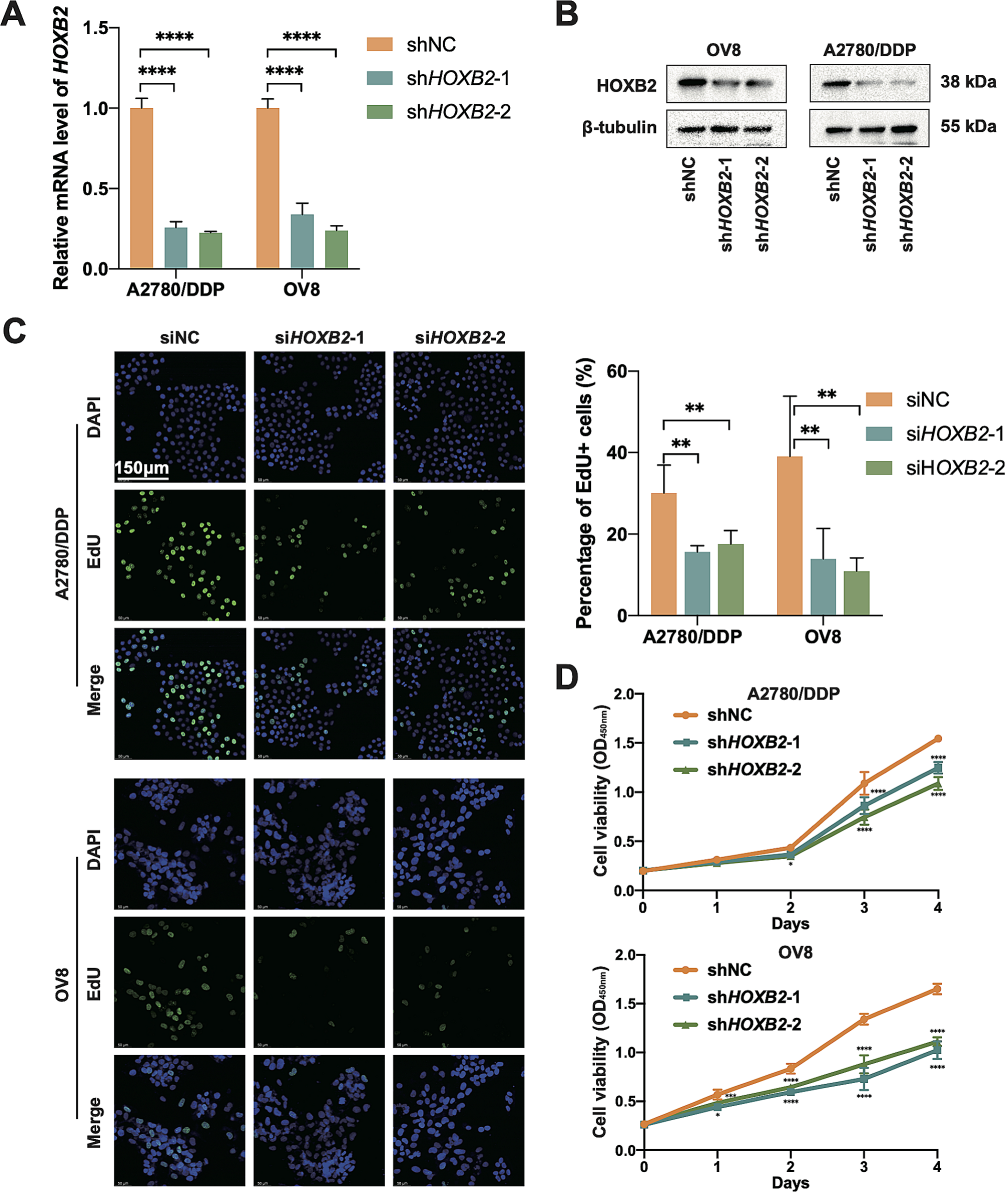
*HOXB2* promotes OV cell growth. **A**–**B**. qPCR and WB results showing the knockdown efficiency of *HOXB2* in A2780/DDP and OV8 cells. **C**. Representative images of the EdU incorporation assay. Scale bar, 150 μm. **D**. Relative cell viability of OV cells transfected with shNC, sh*HOXB2*-1, and sh*HOXB2*-2 lentiviruses. Statistical analyses were performed using a two-way ANOVA. *P < 0.05, **P < 0.01, ***P < 0.001, ***P < 0.0001

**Fig. 3:**
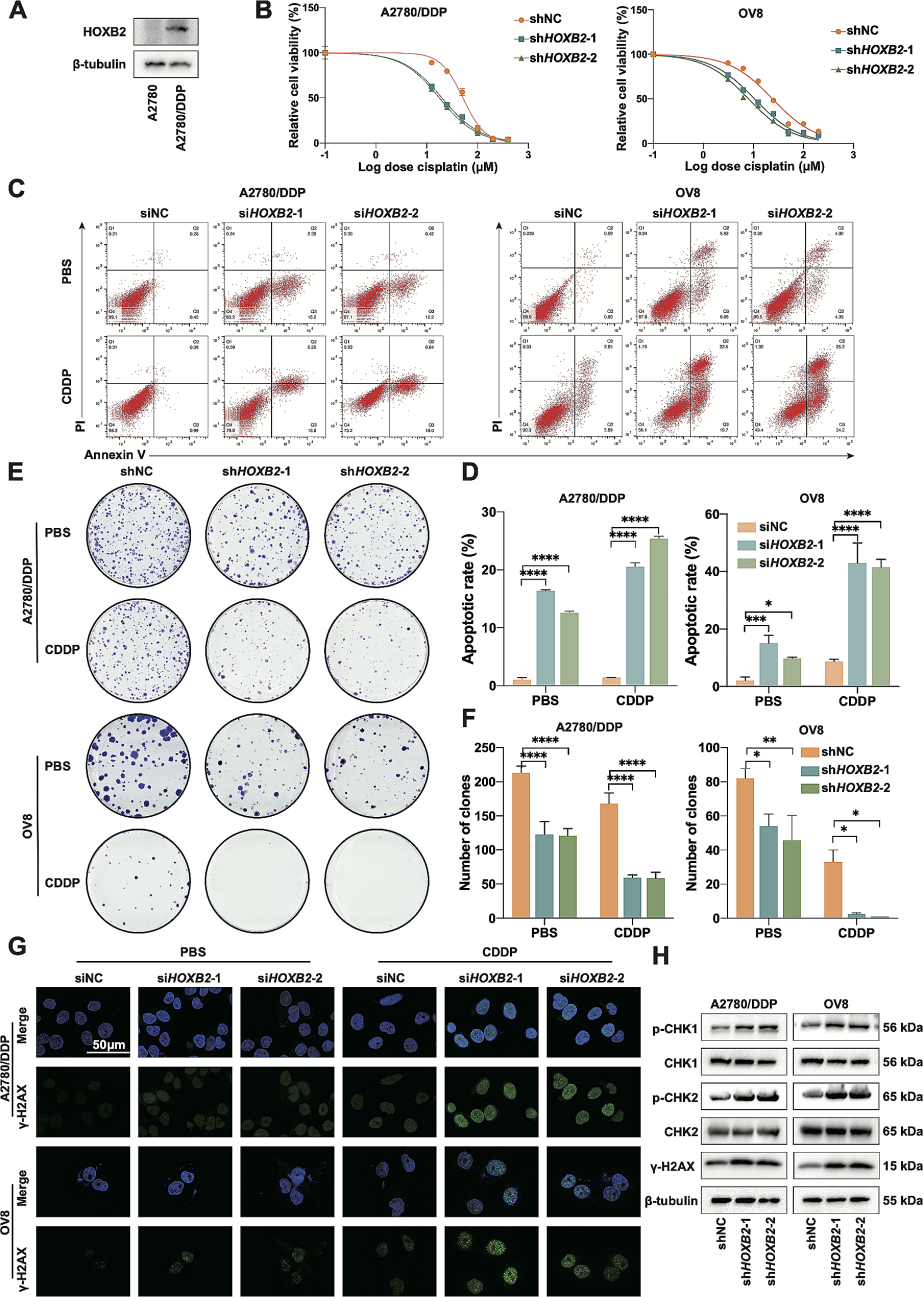
*HOXB2* maintains the cisplatin-resistance of OV cells. **A**. Protein level of HOXB2 in A2780 and A2780/DDP. **B**. Relative cell viability of shNC, sh*HOXB2*-1, and sh*HOXB2*-2 cells at different concentrations of CDDP. Results of A2780/DDP cells: IC50shNC = 51.50 µM, IC50sh*HOXB2*-1 = 19.97 µM, and IC50sh*HOXB2*-2 = 17.76 µM; Results of OV8 cells: IC50shNC = 24.04 µM, IC50sh*HOXB2*-1 = 10.52 µM, and IC50sh*HOXB2*-2 = 7.99 µM. **C**–**D**. Apoptotic assay of siNC, si*HOXB2*-1, and si*HOXB2*-2 cells after treatment with CDDP (20 µM for A2780/DDP and 10 µM for OV8) and equal PBS for 48 h. The statistical significances were calculated using the ANOVA test. **E**–**F**. Colony formation assays of shNC, sh*HOXB2*-1, and sh*HOXB2*-2 cells treated with CDDP (10 µM for A2780/DDP, 5 µM for OV8) and equal PBS. **G**. ICC showed γH2A.X level of siNC, si*HOXB2*-1, and si*HOXB2*-2 cells after treatment of CDDP (20 µM for A2780/DDP, 10 µM for OV8) and equal PBS for 48 h. The statistical significances were calculated using the ANOVA test. **H**. shNC, sh*HOXB2*-1, and sh*HOXB2*-2 cells were treated with CDDP (20 µM for A2780/DDP, 10 µM for OV8) for 48 h, followed by protein extraction and WB. *P < 0.05, **P < 0.01, ***P < 0.001, ***P < 0.0001

To explore the functions and specific mechanisms of *HOXB2* in OV cell growth and resistance, we conducted in vitro experiments on OVCAR8 cells, an OV cell line with relatively high expression of *HOXB2*, and A2780/DDP cells, a platinum-resistant strain of A2780 cells. We constructed stable *HOXB2* knockdown cell lines for both cell types using lentivirus and verified the knockdown efficiency at the mRNA and protein levels (Fig. 2A–B). Subsequently, we evaluated the growth of OV cells after *HOXB2* reduction using the CCK8 cell proliferation assay (Fig. 2D) and plate colony formation assay (Fig. 3E), revealing that the proliferation rate and number of colonies formed in the knockdown group were significantly reduced compared to those in the control group. The cell immunofluorescence assay indicated that the proportion of EdU-positive cells with greater viability in the control group was comparatively higher (Fig. 2C). We used flow cytometry to quantify EdU-positive cells and obtained corresponding results (Supplementary Fig. 1). These results suggested that *HOXB2* promotes OV cell proliferation.

### *HOXB2* maintains the cisplatin-resistance of OV cell

To validate the role of *HOXB2* in OV drug resistance, we compared the expression levels of HOXB2 in A2780 and A2780/DDP cell lines by western blotting and found that the latter had a significantly stronger expression than the former (Fig. 3A). Examination of cisplatin IC50 in OV cells revealed that *HOXB2* knockdown resulted in a considerable decrease in IC50 in both non-resistant OV8 and resistant A2780/DDP cell lines (Fig. 3B). The specific data were as follows: A2780/DDP: IC50shNC = 51.50 µM, IC50sh*HOXB2*-1 = 19.97 µM, and IC50sh*HOXB2*-2 = 17.76 µM; OV8: IC50shNC = 24.04 µM, IC50sh*HOXB2*-1 = 10.52 µM, and IC50sh*HOXB2*-2 = 7.99 µM. Using flow cytometry to detect cell apoptosis, we observed that *HOXB2* knockdown at the gene level alone increased the cell apoptosis rate compared with that in the control group. This trend was further amplified after cisplatin induction (Fig. 3C–D). Results from colony formation assays showed that when cisplatin was added to the two cell lines, the growth of the *HOXB2* knockdown group cells was remarkably inhibited compared to that of the control group cells (Fig. 3E–F). Cisplatin mainly targets tumor cell DNA, and after DNA damage, histone H2A.X is phosphorylated at Ser139. By marking γH2A.X, we noticed more DNA-damaged cells in the knockdown group using immunofluorescence (Fig. 3G). In addition to DNA damage, many proteins, including the core kinases Chk1 and Chk2, are recruited and activated in the DNA damage response (DDR). Therefore, we indirectly examined the severity of tumor cell DNA damage by measuring the expression of γH2A.X and the activation levels of Chk1 and Chk2 by WB, revealing impaired cisplatin tolerance after *HOXB2* downregulation (Fig. 3H). Therefore, our findings support that *HOXB2* promotes the cisplatin-resistance in OV cells.

### Reduction of *HOXB2* sensitizes OV cells to cisplatin in vivo

**Fig. 4:**
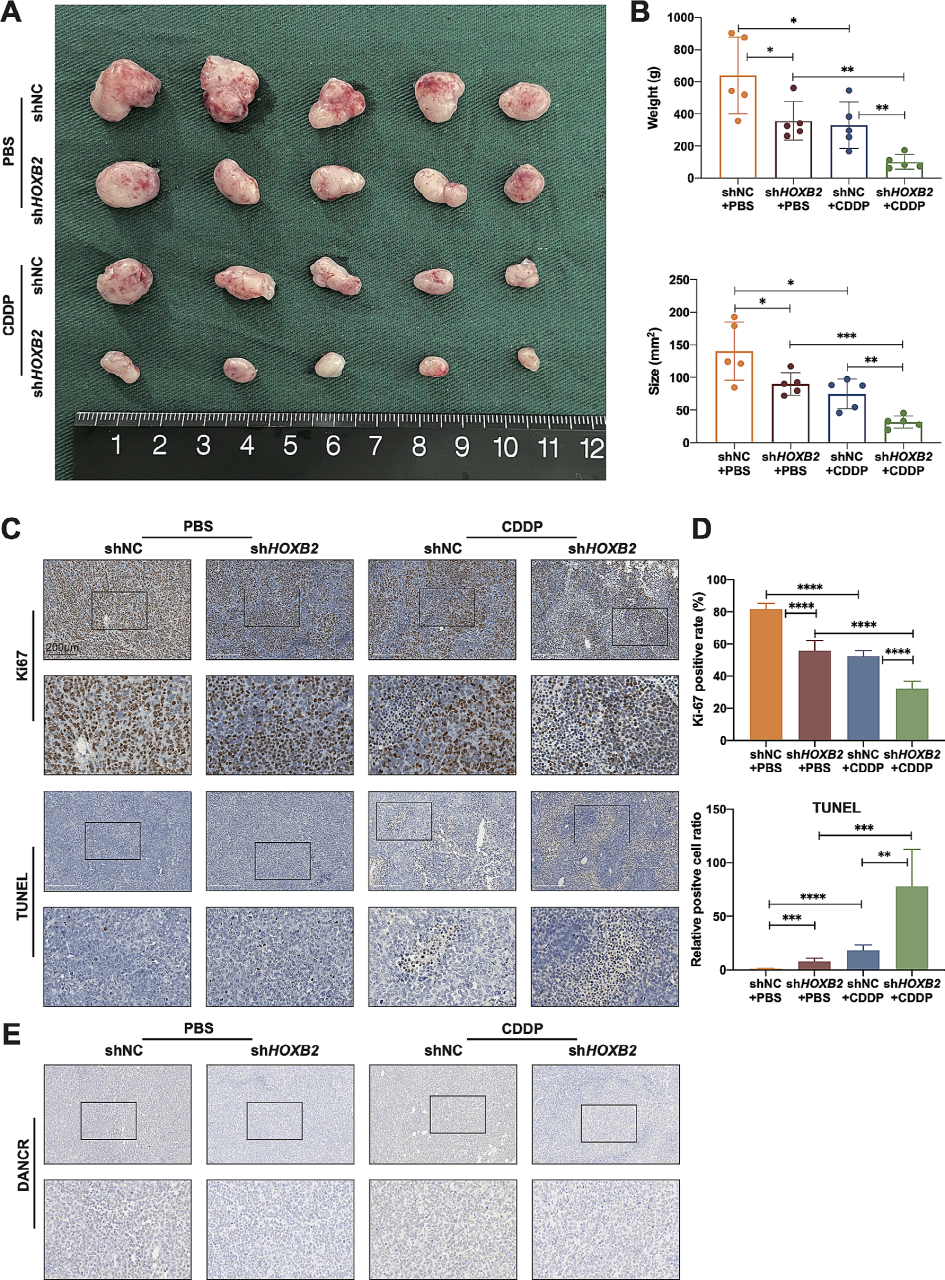
Reduction of *HOXB2* sensitizes OV cells to cisplatin *in vivo.***A**. Subcutaneous xenografts transplanted with A2780/DDP shNC and sh*HOXB2* cells in Balb/c nude mice treated with PBS or 5 mg/kg CDDP. Scale bar, 1 cm. **B**. Statistical analysis of the weight and size of subcutaneous xenografts from the different groups. The P-value between the two groups was calculated using an unpaired *t*-test. **C**–**D**. Represent images (left) and statistical analysis (right) of IHC staining for Ki67 and TUNEL assay in subcutaneous xenografts. The P-value between the two groups was calculated using an unpaired *t*-test. Scale bar, 200 μm. **E**. Results of CISH with DANCR probes in subcutaneous xenografts. *P < 0.05, **P < 0.01, ***P < 0.001, ***P < 0.0001

Previous studies have demonstrated that suppressing *HOXB2* expression in vitro significantly inhibits OV cell proliferation and cisplatin resistance. Therefore, we evaluated the biological functions of *HOXB2 in vivo* using a subcutaneous tumor animal model. Results established that *HOXB2* knockdown minimizes tumor volume and weight while increasing tumor tissue sensitivity to cisplatin (Fig. 4A–B). After fixing and embedding the xenografts in paraffin, immunohistochemical detection of Ki67 and TUNEL tests was performed. The percentage of Ki67-positive cells decreased after *HOXB2* knockdown. We detected more apoptotic cells in the *HOXB2*-inhibited group than in the control group. Both phenomena were more pronounced in the cisplatin injection group (Fig. 4C–D). Using hybridization probes, we detected lower level of DANCR in xenografts after *HOXB2* knockdown (Fig. 4E). These in vivo findings indicated that lowering *HOXB2* could inhibit OV expansion and cisplatin resistance, suggesting that *HOXB2* might be a potential therapeutic target for OV.

### *HOXB2* modulates the expression of ABC transporters and Erk activity

**Fig. 5:**
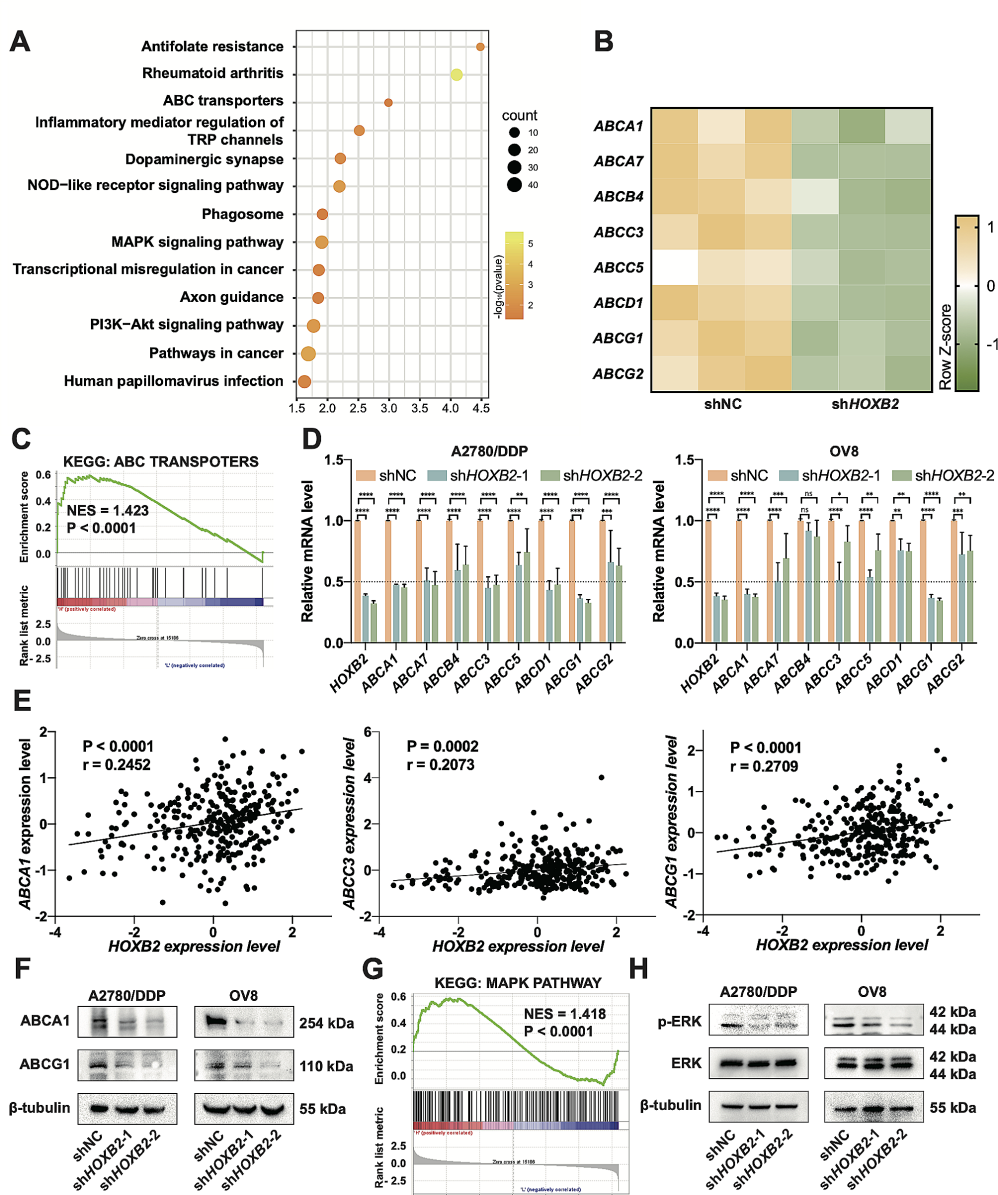
*HOXB2* modulates the expression of ABC transporters and Erk activity. **A**. Functional analysis of the differentially expressed genes from the RNA-Seq results of OV8 shNC cells and sh*HOXB2* cells based on KEGG pathways. **B**. Relative mRNA levels of ABC transporters in OV8 shNC and sh*HOXB2* cells. **C**. Gene set enrichment analysis was performed to compare the OV8 shNC and sh*HOXB2* cells. NES, Normalized enrichment score. **D**. qPCR results showing the expression levels of ABC transporters of shNC, sh*HOXB2*-1, and sh*HOXB2*-2 cells. **E**. Correlation analysis of *HOXB2* with *ABCA1*, *ABCC3*, and *ABCG1* based on the TCGA database. r, Pearson’s correlation coefficient. **F**. WB analysis of ABCA1 and ABCG1 expression in shNC, sh*HOXB2*-1, and sh*HOXB2*-2 cells. **G**. GSEA result of OV8 shNC cells and sh*HOXB2* cells. **H**. WB results of p-MAPK42/44 (Erk1/2) and total Erk levels in shNC, sh*HOXB2*-1, and sh*HOXB2*-2 cells. *P < 0.05, **P < 0.01, ***P < 0.001, ***P < 0.0001

To investigate the specific mechanisms by which *HOXB2* regulates OV cell proliferation and cisplatin resistance, we extracted total RNA from both control and *HOXB2* knockdown groups of cells for RNA-Seq sequencing. The gene expression levels in the sequencing results were standardized and processed, and the DEGs between the two groups were subjected to pathway enrichment analysis. The results showed that the DEGs were primarily related to ATP-binding cassette transporters (ABC transporters), folate resistance, MAPK, PI3K/AKT, and other signaling pathways (Fig. 5A). Previous studies have revealed that the ABC transporter superfamily is crucial for drug resistance in tumor tissues, suggesting that *HOXB2* may affect OV cisplatin resistance by modulating the ABC transporter superfamily. Based on transcriptome sequencing results, we observed that eight genes in this family were downregulated after *HOXB2* reduction, including *ABCA1*, *ABCA7*, *ABCB4*, *ABCC3*, *ABCC5*, *ABCD1*, *ABCG1*, and *ABCG2* (Fig. 5B). Gene set enrichment analysis (GSEA) of the transcriptome sequencing results showed a statistically significant normalized enrichment score (NES) of 1.423 for the ABC transporter pathway (Fig. 5C). Real-time fluorescent quantitative PCR further verified the changes in these genes in the control and stable *HOXB2* knockdown groups in the two cell lines. The shifts in *ABCA1* and *ABCG1* were relatively more evident in the two cell lines (Fig. 5D). TCGA data showed that *HOXB2* was statistically correlated with the transcriptional expression of *ABCA1*, *ABCC3*, and *ABCG1* (Fig. 5E). Based on the above results and the existing literature, we focused on the genes *ABCA1* and *ABCG1* and verified their downregulation after *HOXB2* suppression at the protein level (Fig. 5F).

On the other hand, we observed changes in some tumor-related signaling pathways following *HOXB2* downregulation. GSEA displayed an NES of 1.418, with statistical significance for the MAPK signaling pathway (Fig. 5G). Western blotting proved that reducing *HOXB2* caused a decline in the phosphorylation of MAPK 42/44 (Erk1/2) (Fig. 5H), implying that *HOXB2* may elevate OV cell growth by activating the ERK signaling pathway.

### *HOXB2* strengthens the proliferation and cisplatin resistance of OV cells via DANCR

**Fig. 6:**
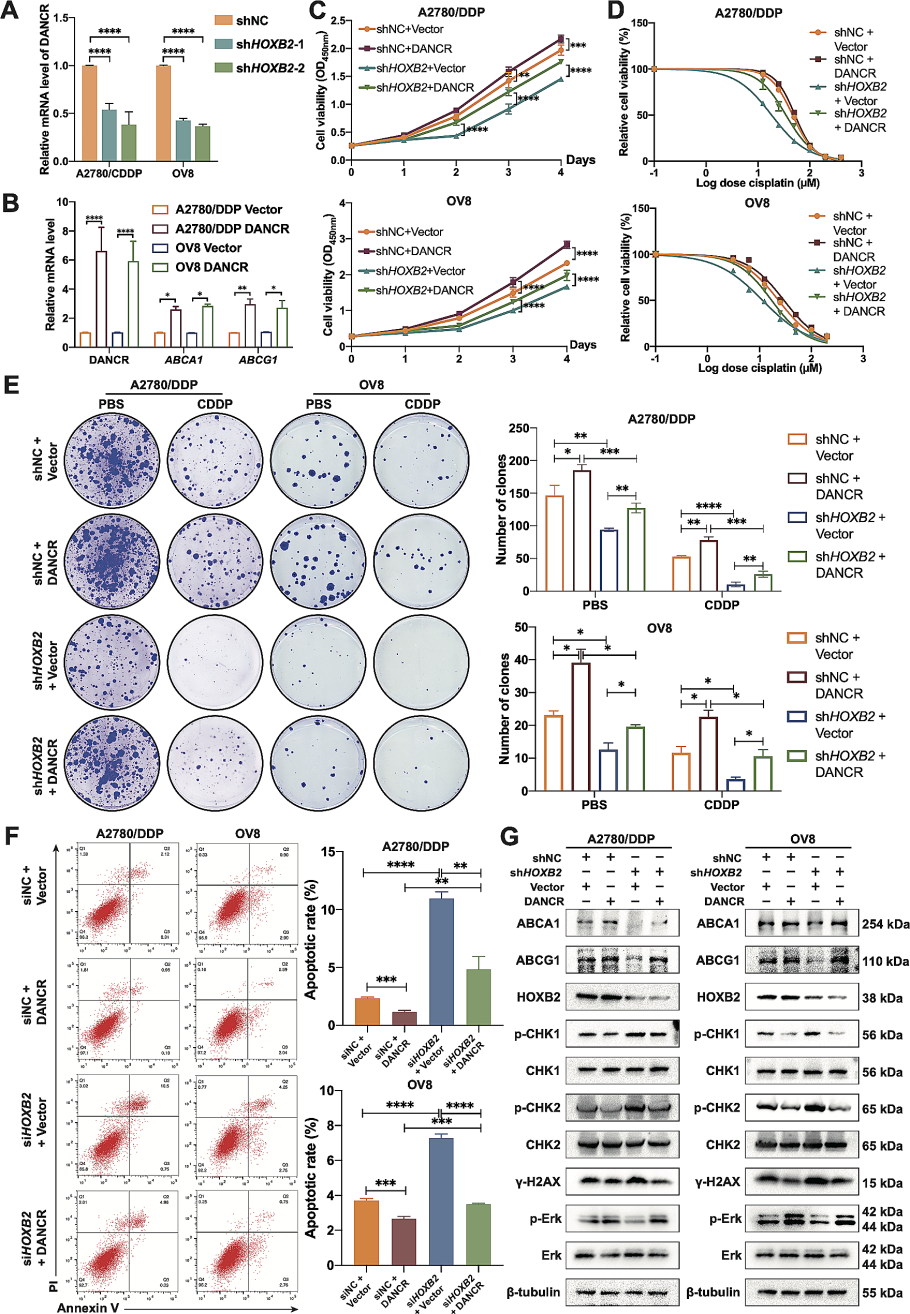
*HOXB2* promotes the proliferation and cisplatin resistance of OV cells via DANCR. **A**–**B**. qPCR results showing the mRNA levels of the target genes. **C**. Relative cell viability of shNC + Vector, shNC + DANCR, sh*HOXB2* + Vector, and sh*HOXB2* + DANCR cell groups. Statistical significance was calculated using two-way ANOVA. **D**. Relative cell viability of shNC + Vector, shNC + DANCR, sh*HOXB2* + Vector, sh*HOXB2* + DANCR cells at different concentrations of CDDP. **E**. Colony formation assays of shNC + Vector, shNC + DANCR, sh*HOXB2* + Vector, sh*HOXB2* + DANCR cells treated with CDDP (10 µM for A2780/DDP, 5 µM for OV8) and equal PBS. The P-value between the two groups was calculated using an unpaired *t*-test. **F**. Apoptotic assay of siNC + Vector, siNC + DANCR, si*HOXB2* + Vector, si*HOXB2* + DANCR cells after treatment of CDDP (20 µM for A2780/DDP, 10 µM for OV8) and equal PBS for 24 h. The P-value between the two groups was calculated using an unpaired *t*-test. **G**. Western blotting showed the target protein level of shNC + Vector, shNC + DANCR, sh*HOXB2* + Vector, sh*HOXB2* + DANCR cells after treatment of CDDP (20 µM for A2780/DDP, 10 µM for OV8) for 48 h. *P < 0.05, **P < 0.01, ***P < 0.001, ***P < 0.0001

Previous studies have demonstrated that *ABCA1* can be regulated by various lncRNAs. By examining the expression of 18 lncRNAs included in the above-mentioned study [21] using RNA-seq results, we found that only DANCR decreased with the downregulation of *HOXB2* expression. QPCR was used to confirm the changes in DANCR RNA expression in the control group and stable *HOXB2* knockdown group in the two cell lines, and the trends were consistent with the sequencing data (Fig. 6A). Consequently, we hypothesized that *HOXB2* promotes downstream transcription of *ABCA1* by influencing the level of DANCR. Moreover, since lncRNAs exhibit complex and precise regulatory functions in gene expression and participate in multiple biological processes in cells, we speculated whether DANCR can also promote *ABCG1* expression, a member that has synergistic functions with *ABCA1*. We overexpressed DANCR in A2780/DDP and OV8 cell lines and established that the mRNA levels of *ABCA1* and *ABCG1* increased with increasing DANCR expression (Fig. 6B). After overexpressing DANCR, the protein levels of ABCA1 and ABCG1 also increased, and this phenomenon was more apparent in *HOXB2*-reduced cells, possibly because their expression in the control group was more prone to saturation (Fig. 6G). We then designed experiments to test whether *HOXB2* mediated platinum resistance in OV cells by affecting the expression of *ABCA1* and *ABCG1* through DANCR. Cisplatin IC_50_ measurements showed that DANCR overexpression partially restored the drug tolerance caused by *HOXB2* suppression (Fig. 6D). Similarly, flow cytometry results disclosed that DANCR overexpression significantly reduced cisplatin-induced apoptosis in both cell lines (Fig. 6F). We then tested the degree of DNA damage by WB and confirmed that rescuing DANCR expression alleviated the DNA damage caused by cisplatin and reduced the accompanying DDR reaction (Fig. 6G).

Meanwhile, existing studies have reported that DANCR can affect the activation of the ERK pathway. Therefore, we hypothesized that DANCR might have a similar function in OV cells and that *HOXB2* affectd the activation of ERK signaling to accelerate tumor growth. Both cell proliferation and clone formation assays were employed to validate that the DANCR upregulation partially repaired the cell viability diminished by *HOXB2* decline (Fig. 6C and E). At the protein level, we observed that restoring DANCR expression could revive the phosphorylation of Erk1/2, thus supporting our hypothesis (Fig. 6G).

## Discussion

Platinum resistance is a common hindrance that cannot be circumvented during OV treatment. Decades-long worldwide efforts have been dedicated to relieving patient distress. Various molecular mechanisms, including multidrug resistance (MDR) [22], abnormal DNA damage repair [23], and cell cycle regulation, contribute to OV drug resistance. Through bioinformatic analysis of TCGA, GTEx, and GEO databases and additional verification using clinical samples, an upregulation of HOXB2 was identified in OV, especially in the platinum-resistant cases, and this upregulation was found to be associated with poor prognosis. Our in vivo and in vitro investigations further supported this finding by illustrating the role of *HOXB2* in enhancing ovarian cancer cell proliferation and resistance to cisplatin. The expression of *HOXB2* can guide the development of personalized treatment strategies, ultimately improving patient survival rates. The expression of genes is regulated by multiple factors, and the translation efficiency and accuracy of post-transcriptional mRNA can be controlled by factors such as miRNA. In order to explore the relationship between miRNA and HOXB2 expression, we conducted predictions through an online website (https://rnasysu.com/encori/). In the results, we predicted that hsa-miR-145-5p may regulate the expression of *HOXB2*. The hsa-miR-145-5p has been reported to be highly expressed in ovarian cancer and associated with poor prognosis in ovarian cancer. Further research is needed to confirm the relationship between hsa-miR-145-5p and HOXB2 in ovarian cancer.

Numerous HOX family genes, including *HOXB2*, have been reported to be abnormally expressed in tumors and to accelerate carcinogenesis. A previous study identified *HOXB2* as a retinoic acid signaling target and predictor of pancreatic cancer [24]. In esophageal squamous cell carcinoma, *HOXB2* transcriptionally induces cancer cell stemness [25]. Through bioinformatic analysis of TCGA, GTEx, and GEO databases and additional verification using clinical samples, we identified an upregulation of *HOXB2* in OV, particularly the platinum-resistant ones, which was correlated with poor prognosis. This finding was further corroborated by our in vivo and in vitro investigations, which demonstrated the function of *HOXB2* in promoting OV cell growth and cisplatin tolerance.

In the subsequent mechanistic exploration, we conducted RNA-Seq and discovered that *HOXB2* regulates several ABC transporters. ABC transporters translocate multiple substrates, including nutrients, metabolites, and drugs, across cellular membranes using ATP hydrolysis [26]. The enhanced efflux of drugs due to overexpression of the ABC family is an important mechanism of multidrug resistance in cancer chemotherapy [27, 28]. We observed that *HOXB2* has the potential to influence the ABC transporter members *ABCA1*, *ABCA7*, *ABCB4*, *ABCC3*, *ABCC5*, *ABCD1*, *ABCG1*, and *ABCG2* transcriptionally. Through further experiments and bioinformatics analysis, we focused on the regulatory function of *HOXB2* in *ABCA1* and *ABCG1*. These two genes are widely recognized in atherosclerosis because of their role in cholesterol transportation [29, 30]. Regarding cancer drug resistance, certain findings in OV indicate that downregulation of *ABCA1* re-sensitizes cisplatin-resistant OV cells [31]. *ABCG1* has been reported to be hypermethylated in decitabine-treated OV patients with longer survival [32]. We revealed that DANCR, among several lncRNAs that have been reported to regulate the expression of *ABCA1* [21], could modulate both *ABCA1* and *ABCG1*. Overexpression of DANCR restored the decrease in *ABCA1* and *ABCG1* expression caused by suppression of *HOXB2* and subsequent cisplatin tolerance reduction. Previous studies have confirmed that DANCR acts as a sponge for several microRNAs (miR-33a-5p [33, 34] and miR-758-3p) [35, 36] to promote *ABCA1* expression. However, the mechanism in DANCR regulating *ABCG1* remains unknown and needs to be uncovered.

Our results showed that *HOXB2* could activate the ERK signaling pathway via lncRNA DANCR. A study on cervical cancer supports this finding that DANCR mediates the ERK/SMAD pathway via miR-665 [37]. ERK signaling is a well-characterized MAPK pathway known for its potential to promote uncontrolled cancer cell growth. Moreover, this highly activated pathway also participates in cisplatin resistance in tumors, including breast [38], lung [39], and ovarian cancers [40, 41], with context-dependent mechanisms. For instance, ERK signaling enhances glycolysis and the non-oxidative pentose phosphate pathway to increase protective nucleotide metabolism [38]. In OV, it phosphorylates mitogen-activated protein kinase phosphatase-1 (MKP-1) to induce drug resistance [40]. In this study, we delineated that the *HOXB2*-promoted ERK pathway accelerated the proliferation of OV cells and might further strengthen cisplatin tolerance.

In conclusion, our research is the first to demonstrate that *HOXB2* is upregulated in OV and is correlated with platinum resistance. Dysregulated *HOXB2* promotes ABC transporter members and ERK pathway through DANCR to enhance cancer chemotherapy tolerance and cell growth. Although the exact mechanisms of the *HOXB2*-DANCR-downstream effectors axis have not been fully explored, our findings suggest that *HOXB2* might serve as a potential target for OV treatment.

### Electronic supplementary material

Below is the link to the electronic supplementary material.

Supplementary Fig. 1. Results of flow cytometry after EdU incorporation in OV cells transfected with siNC, si*HOXB2*-1, and si*HOXB2*-2 lentiviruses. Statistical analyses were performed using a two-way ANOVA. *P < 0.05, **P < 0.01, ***P < 0.001, ***P < 0.0001.
