# Supplementary figures and images for: HOXB2 promotes cisplatin resistance by upregulating lncRNA DANCR in ovarian cancer
Source: J Ovarian Res. 2024 Jun 8;17:124. doi: 10.1186/s13048-024-01424-1 (PMC11161928; doi:10.1186/s13048-024-01424-1)

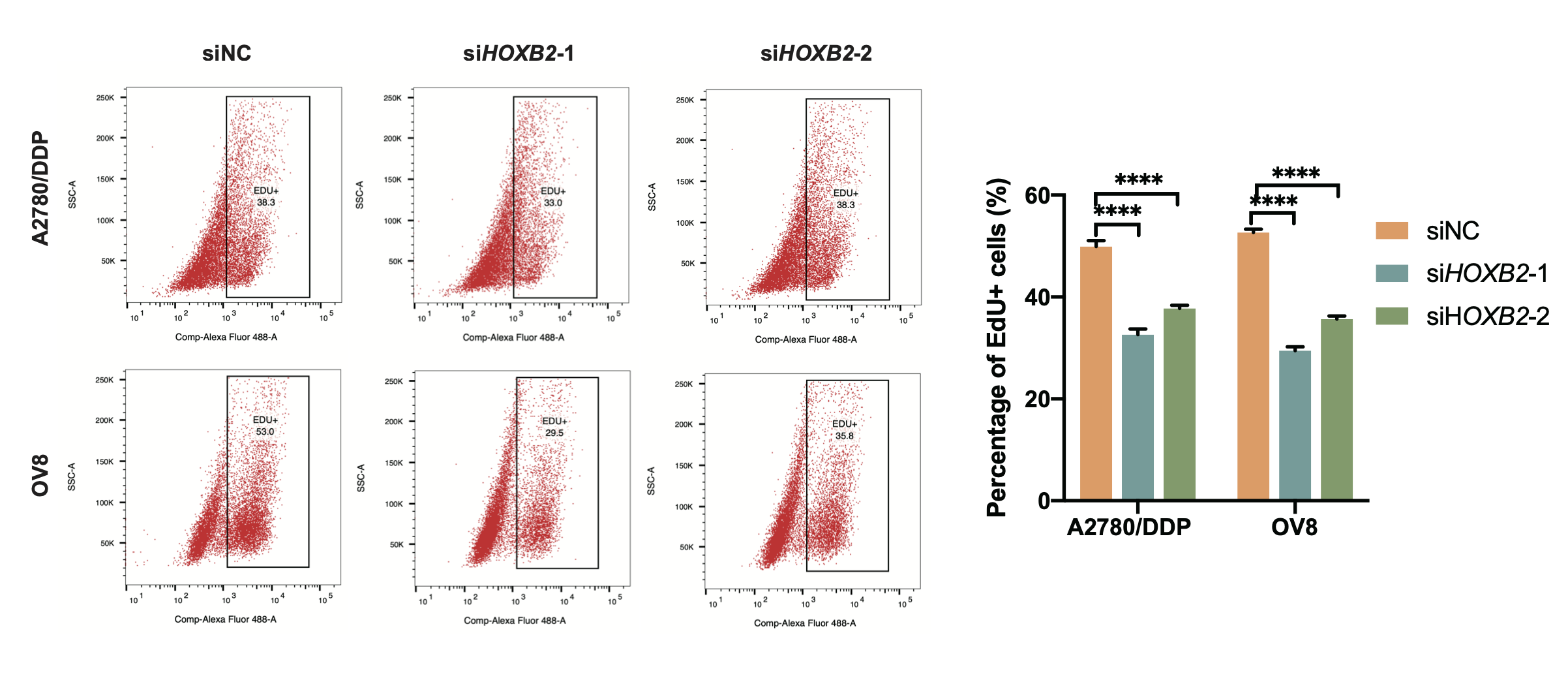

Supplement: Supplementary file 1 — Supplementary Fig. 1. Results of flow cytometry after EdU incorporation in OV cells transfected with siNC, siHOXB2-1, and siHOXB2-2 lentiviruses. Statistical analyses were performed using a two-way ANOVA. *P < 0.05, **P < 0.01, ***P < 0.001, ***P < 0.0001. [file 13048_2024_1424_MOESM1_ESM.tiff]
